# Bleeding Risk With Antiplatelets and Bruton's Tyrosine Kinase Inhibitors in Patients With Percutaneous Coronary Intervention

**DOI:** 10.1016/j.jscai.2023.100608

**Published:** 2023-03-06

**Authors:** Alan Mendez-Ruiz, Izidore S. Lossos, Mauricio G. Cohen

**Affiliations:** a Department of Medicine, University of Miami Miller School of Medicine, Miami, Florida; b Division of Hematology, Department of Medicine and Sylvester Comprehensive Cancer Center, University of Miami, Miami, Florida; c Department of Molecular and Cellular Pharmacology, University of Miami Miller School of Medicine, Miami, Florida; d Cardiovascular Division, Department of Medicine, University of Miami Miller School of Medicine, Miami, Florida

**Keywords:** antiplatelet, bleeding, Bruton's tyrosine kinase inhibitor, percutaneous coronary intervention

## Abstract

Bruton's tyrosine kinase inhibitors (BTKi), known for their off-target antiplatelet effects and increased bleeding risk, have become more frequently used in patients with cancer with lymphoid malignancies. When encountering patients on BTKi who also require antiplatelet therapies, particularly dual-antiplatelet therapy that is a well-established treatment after percutaneous coronary intervention (PCI), the potential increase in bleeding risk raises concern. The development of new-generation BTKi with a higher selectivity, such as acalabrutinib and zanubrutinib, could be an answer to this conundrum, but bleeding still occurs. It is important that the interventional cardiologist becomes familiar with agents that could affect the management of antiplatelet therapy in patients treated with PCI.

## Highlights

• Bruton's tyrosine kinase inhibitors (BTKi) have off-target antiplatelet effects
• New covalent BTKi have not shown superiority in reducing major bleeding adverse events
• The combination of BTKi with antiplatelets suggests an increased bleeding risk
• Further studies are needed to assess the interaction between BTKi and antiplatelets and their combined bleeding risk
• Cardiology and hematology collaboration is warranted for optimal care of PCI patients on BTKi

## Introduction

Ibrutinib, the first covalent Bruton's tyrosine kinase inhibitor (BTKi), was first tested in clinical trials in patients with chronic lymphocytic leukemia (CLL) in 2009. It was approved by the US Food and Drug Administration (FDA) in 2013.^1^ Multiple toxicities have been reported with its use.^2^ Bleeding is a well-known adverse event (AE) reported in up to 55% of the patients taking ibrutinib.^3^ The underlying mechanism of ibrutinib-associated bleeding relates to its off-target inhibition of kinases, interfering with normal platelet signaling, adhesion, and aggregation.^4^, ^5^, ^6^ Initially, ibrutinib was contraindicated in patients treated with warfarin owing to excessive bleeding risk and raised the issue whether BTKi agents can be safely administered in patients requiring antiplatelet therapy.^5^

Newer generation of covalent BTKi, acalabrutinib and zanubrutinib, were designed with a greater selectivity for BTK with the premise of decreasing associated toxicities.^7^^,^^8^ Acalabrutinib was approved by the FDA and European Medicines Agency for the treatment of patients with CLL/small lymphocytic lymphoma (SLL), and it was also approved by the FDA for patients with pretreated mantle cell lymphoma (MCL).^9^ Zanubrutinib was approved by the FDA for patients with pretreated MCL, pretreated marginal zone lymphoma, and Waldenström macroglobulinemia, and was approved by the European Medicines Agency for patients with pretreated Waldenström macroglobulinemia.^10^

The aim of this article is to inform interventional cardiologists about the bleeding risk associated with new-generation covalent BTKi and concomitant use of antiplatelet agents that could affect the antiplatelet management of patients on BTKi who required percutaneous coronary intervention (PCI).

## BTKi-associated antiplatelet mechanism

The underlying antiplatelet mechanism of covalent BTKi has been associated with the inhibition of off-target tyrosine kinases interfering with normal platelet function and can be summarized as follows (Central Illustration):

1. BTKi have been observed to inhibit downstream signaling of platelet collagen receptor glycoprotein (GP) VI. GPVI is responsible for platelet activation and aggregation in response to collagen and collagen-related peptide exposures.^5^ Comparative studies have shown that acalabrutinib and zanubrutinib are less potent inhibitors of GPVI-mediated platelet aggregation than ibrutinib. Strong inhibition of GPVI has been associated with increased closure time on platelet-function analyzer. Ibrutinib and acalabrutinib at higher blood concentrations have shown to increase the platelet-function analyzer closure time.^11^
2. BTKi inhibit tyrosine kinase–linked GPIb platelet receptor for von Willebrand factor (vWF). While the main role of GPVI is initiating platelet intracellular signaling, GPIb mediates platelet adhesion to collagen through vWF.^12^ Inhibition of the 2 receptors has been correlated with the use of ibrutinib. In vitro human blood samples treated with the same concentrations of ibrutinib and zanubrutinib demonstrated that zanubrutinib-treated platelets adhered to and formed a stable thrombi on collagen type 1, vWF, and fibrinogen under high shear flow conditions over a 6-minute period, whereas ibrutinib-treated platelets showed a significant decrease in thrombus formation with a reduced thrombus volume under similar circumstances (*P* < .001).^13^
3. Ibrutinib, but not zanubrutinib, has shown to induce shedding of integrin αIIbβ3,^13^ which has a key role in platelet adhesion and aggregation, and outside-in signaling to provide positive feedback for platelet-activating stimuli for clot retraction and stability.^12^ Integrin αIIbβ3 expression on platelets derived from ibrutinib-treated patients with CLL was significantly lower compared with that from healthy controls, untreated patients, or zanubrutinib-treated patients (*P* < .001). Furthermore, ibrutinib-treated CLL platelets showed reduced thrombus growth on type 1 collagen over time compared with those from healthy controls, untreated CLL, or zanubrutinib-treated patients (*P* < .01).^13^
4. Low concentrations of acalabrutinib and ibrutinib have been shown to inhibit C-type lectin-like receptor 2 downstream signaling involved in platelet aggregation and thrombus stability after platelet adhesion.^14^ In vivo studies showed that mice with depletion of C-type lectin-like receptor 2 had significantly prolonged bleeding time compared with control (*P* < .01).^15^
5. Tec, a member of the family of tyrosine kinase, has also been found to be inhibited by ibrutinib. Tec is involved in the regulation of phospholipase C (PLC)γ2 downstream of collagen receptor GPVI.^6^ PLCγ2 promotes the hydrolyzation of phosphatidylinositol 4,5-bisphosphate to diacylglycerol and inositol trisphosphate in platelets. The increase in platelet diacylglycerol and inositol trisphosphate subsequently upregulate protein kinase C activity, thromboxane A2 content, and calcium concentration (Ca2+).^16^ Decreased inhibition of Tec kinases by acalabrutinib and zanubrutunib was proposed as a potential mechanism for decreased bleeding rates observed with these BTKi in comparison with that of ibrutinib.^5^

**Central Illustration:**
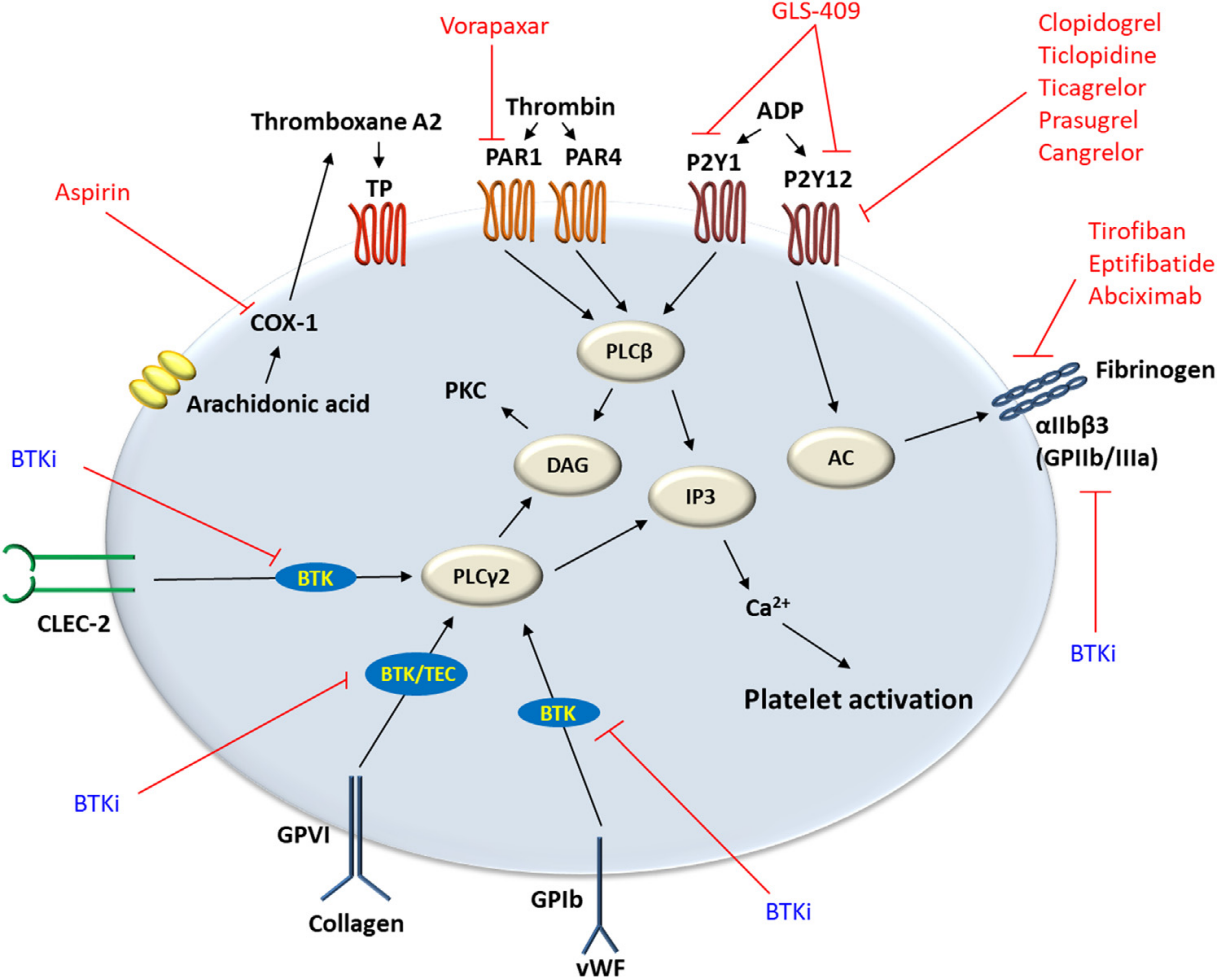
**Schematic representation of platelet receptors, major platelet activation pathways, and inhibitory agents.** Cyclooxygenase (COX)-1 catalyzes the conversion of arachidonic acid to thromboxane A2, which activates thromboxane prostanoid (TP) receptor. COX-1 is inhibited by aspirin. Thrombin activates protease-activated receptors (PARs; PAR1, and PAR4) involved in phospholipase C (PLC)β activation for the formation of inositol-1,4,5-triphosphate (IP3) and diacylglycerol (DAG). This promotes increase in intracellular calcium and upregulation of protein kinase (PK) C. PAR1 is inhibited by vorapaxar. Adenosine diphosphate (ADP) receptor, P2Y1, is involved in PLCβ activation pathway. P2Y12 downregulates adenylyl cyclase (AC) for sustained platelet aggregation through integrin αIIbβ3 (GPIIb/IIIa). GLS-409 is a P2Y1 and P2Y12 inhibitor. Examples of P2Y12 inhibitors are clopidogrel and ticagrelor. Tirofiban, eptifibatide, and abciximab are αIIbβ3 antagonists. Bruton's tyrosine kinase inhibitor (BTKi), ibrutinib, has been observed to induce shedding of integrin αIIbβ3. GPVI collagen receptor, GPIb von Willebrand factor (vWF) receptor, and C-type lectin-like receptor (CLEC)-2 are involved in platelet activation through PLCγ2. Ibrutinib has shown to be a more potent inhibitor of GPVI-linked tyrosine kinases than acalabrutinib and zanubrutinib. Ibrutinib has also been observed to inhibit GPIb downstream signaling. Low concentrations of acalabrutinib and ibrutinib have been reported to inhibit CLEC-2–associated platelet activation.

## Bleeding risk with BTKi

In a phase 2 study in 2015, Lipsky et al^3^ reported that 47 (55%) of 85 patients taking ibrutinib developed bleeding AE. In the phase 3 RESONATE trial to evaluate ibrutinib’s safety and efficacy in comparison with ofatumumab in previously treated patients with CLL, bleeding AE were more commonly seen in patients treated with ibrutinib (44% vs 12%), with a median treatment exposure of 8.6 months (Supplemental Table S1). Most bleeding AE were minor (grades 1-2, according to the National Cancer Institute Common Terminology Criteria for Adverse Events, version 4.0), whereas major bleeding AE (grade ≥3) occurred in 1% of the cases.^17^ This is lower than the 4% major bleeding AE rate observed in the RESONATE 2 trial for patients treated with ibrutinib.^18^ In a systematic review and meta-analysis published in 2017, the overall pooled bleeding annual incidence in patients treated with ibrutinib was 20.8 per 100 patient-years (95% CI, 19.1-22.1) vs 11.6 per 100 patient-years in patients treated with alternative therapies (95% CI, 9.1-14.4). The pooled incidence of grade ≥3 bleeding in patients receiving ibrutinib was 2.76 per 100 patient-years (95% CI, 2.07-3.53).^19^

Regarding acalabrutinib, ASCEND and ELEVATE-TN were pivotal phase 3 trials that evaluated safety and efficacy of acalabrutinib in comparison with those of the standard treatments. In the ASCEND trial, bleeding occurred more commonly with acalabrutinib compared with standard treatments (idelalisib plus rituximab or bendamustine plus rituximab) (26% vs 7%; *P* < .001). However, grade ≥3 bleeding occurred in 2 patients (1%) treated with acalabrutinib monotherapy vs 3 patients (2%) (*P* = .65) treated with standard therapies.^20^ In the ELEVATE-TN trial, Sharman et al.^21^ reported bleeding AE in 76 (43%) of the 178 patients receiving acalabrutinib-obinutuzumab, 70 (39%) of the 179 patients on acalabrutinib monotherapy, and 20 (12%) of the 169 patients on obinutuzumab-chlorambucil. Grade ≥3 bleeding AE were only reported in 3 cases in the acalabrutinib-obinutuzumab group, and 3 in the acalabrutinib-monotherapy group.^21^ An open-label randomized, noninferiority, phase 3 trial in 533 patients with CLL compared acalabrutinib with ibrutinib. At the data cutoff, 124 acalabrutinib-treated patients (46.3%) and 109 ibrutinib-treated patients (41.1%) remained on the treatment. Bleeding AEs were less frequently reported with acalabrutinib (38% vs 51.3%; *P* < .05) after a median follow-up of 40.9 months. Major bleeding rates were identical with acalabrutinib and ibrutinib with 12 cases (5%) and 14 cases (5%) in each group, respectively (Supplemental Table S1).^22^

As for zanubrutinib, overall bleeding AE were more commonly seen with its use when compared to standard therapies (45% vs 11%; *P* < .01) for patients with untreated CLL/SLL after a median treatment duration of 26 months (Supplemental Table S1). Major bleeding AE were comparable among both groups (zanabrutinib 4% vs bendamustine plus rituximab 2%; *P* = .19); however, grade 5 bleeding was only seen in 1 patient treated with zanabrutinib (<1%).^23^ The ASPEN trial evaluated zanabrutinib vs ibrutinib in patients with Waldenström macroglobulinemia. Overall bleeding was similar between groups (zanabrutinib 4.4 per 100 person-months vs ibrutinib 7.0 per 100 person-months; *P* = .08), in addition to bleeding grade of ≥3 (zanabrutinib 6% vs ibrutinib 9%; *P* = .39).^8^ The ALPINE study is an ongoing randomized phase 3 trial comparing zanabrutinib vs ibrutinib in patients with refractory/relapsed CLL/SLL. In the first interim analysis, major bleeding rates were slightly lower with zanubrutinib (2.9%) than those with ibrutinib (3.9%) at a median follow-up of 15 months.^24^

Despite the trial-derived data suggesting antiplatelet effects of BTKi, there are limited data concerning the ramifications to bleeding risk when combined with dual-antiplatelet therapy (DAPT) commonly used in cardiovascular practice.

## Bleeding risk with BTKi and antiplatelet drugs

The antiplatelet properties of BTKi have raised the question whether these agents are safe in patients with cardiovascular disease treated with DAPT. In vitro data have suggested that ibrutinib and acalabrutinib at clinically relevant doses can potentiate the effects of other antiplatelet therapies.^4^^,^^12^ Series et al^4^ conducted a study to evaluate the BTKi inhibitory effect on platelet aggregation using platelet-rich plasma of healthy donors. Their antiaggregation effect was also assessed when used with indomethacin or cangrelor. Although ibrutinib showed an inhibitory effect of >50% of maximal platelet aggregation, acalabrutinib had weak or no effect. However, when combined with either indomethacin or cangrelor, both medications exhibited a significant decrease in platelet aggregation, thus suggesting the potentiation of cyclooxygenase inhibition and P2Y12 antagonism.^4^ This raises a concern on whether acalabrutinib could be a safe alternative for ibrutinib in patients requiring antiplatelet therapy. Zanubritinib’s antiplatelet properties have not been evaluated with the concomitant use of antiplatelet agents.

At a clinical level, the lack of inclusion of patients at risk for bleeding in clinical trials and the overall tendency to group anticoagulants and antiplatelets in a single group limit further evaluation of the true bleeding effect association between antiplatelet therapy and BTKi. In an open-label phase 2 registration trial of ibrutinib for patients with MCL, bleeding AE were reported more frequently in patients receiving an antithrombotic agent (69% any grade; 8% grade 3-4) than that in those who did not (28% any grade, 4% grade 3-4). Among the 7 patients who experienced grade ≥3 bleeding AE, 5 had been taking aspirin or nonsteroidal anti-inflammatory drugs before the AE.^25^ Similarly, Jones et al^26^ reported that 4 of 8 patients with CLL with major bleeding during ibrutinib treatment had also received aspirin or nonsteroidal anti-inflammatory drugs. However, in a safety pooled analysis of 4 randomized controlled studies that included ibrutinib and standard therapies, the association between major hemorrhage and the use of anticoagulant and/or antiplatelet agents was not significant for either group.^27^

As for acalabrutinib and zanubrutinib, there is a very limited description of the concomitant use of antithrombotic agents in clinical trials. In the ELEVATE-TN study, 4 of 6 patients with reported grade ≥3 bleeding were also given antithrombotic agents of which 2 patients had received aspirin and 1 patient clopidogrel. One patient was discontinued from the acalabrutinib-obinutuzumab treatment group owing to a perceived increase in bleeding risk when DAPT was added for a non–ST myocardial infarction requiring a stent.^21^ In the SEQUOIA trial, 4 patients treated with zanubrutinib and experiencing major bleeding were concurrently treated with antithrombotic therapies, including aspirin.^23^

Real-world data with BTKi and DAPT commonly used in cardiology are lacking. Although the effects of BTKi and antiplatelets may be additive as suggested, the underlying true effect remains unknown.

## BTKi antiplatelet properties on the atherosclerotic plaque

It has been reported that the collagen associated with the atherosclerotic plaque structurally differs from that of healthy connective tissue.^28^ Although injured plaque exposes structurally diverse collagen types 1 and 3 fibers that induce platelet aggregation under static and flow conditions mainly through GPVI, native collagen does so by GPVI and collagen receptor integrin α2β1.^12^^,^^28^ Thus, BTKi selective inhibition for GPVI and sparing of integrin α2β1 could prevent plaque-stimulated aggregation, whereas platelet adhesion to native collagen dependent on integrin α2β1 remains unaffected, which is essential for physiologic hemostasis.^28^^,^^29^ This was demonstrated by an in vitro and ex vivo study, in which ibrutinib and newer-generation BTKi acalabrutinib and ONO/GS-4059 blocked GPVI-dependent static platelet aggregation in blood exposed to human plaque homogenate and collagen. Furthermore, BTKi prevented platelet thrombus formation on human atherosclerotic plaque homogenate and plaque tissue sections from arterially flowing blood, whereas integrin α2β1 and vWF-dependent platelet adhesion was not affected.^28^ Ultimately, it was reported that ibrutinib at a low dose (140 mg) daily or on alternate days for 1 week caused full suppression of atherosclerotic plaque-induced platelet aggregation under static and flow conditions and was more effective than aspirin (100 mg/d).^28^^,^^29^ BTKi have been proposed as a promising oral antiplatelet drug focused on reducing atherothrombosis.^28^

## Conclusions

Over the past decade, BTKi have become increasingly used for treatment of hematologic malignancies and exert antiplatelet effects with a mechanism of action that is different than that of aspirin and P2Y12 inhibitors, agents commonly used in cardiovascular medicine.

Despite the increased selectivity of newer BTKi, associated bleeding AE continue to occur in more than a quarter of patients treated with these agents. Head-to-head comparisons between ibrutinib and acalabrutinib suggest a reduction in minor bleeding AE with acalabrutinib, but similar major bleeding AE rates are observed with both therapies (Supplemental Table S1). Zanubrutinib has not demonstrated a clear advantage in reducing bleeding AE either.

It is unknown whether BTKi can prevent stent thrombosis, allow early de-escalation of DAPT after PCI, or have a role in the treatment of atherosclerotic cardiovascular disease, but it is clear that these agents increase bleeding risk, which can be exaggerated in patients on DAPT after PCI. Large trials assessing vorapaxar, an antiplatelet that blocks the thrombin receptor, was associated with increased bleeding risk when added to patients already on DAPT.^30^ Therefore, it is plausible that the additive antiplatelet effect of BTKi may also increase bleeding risk in this population, raising serious safety concerns.

Studies focusing on BTKi and commonly used cardiovascular antiplatelet agents, such as aspirin and P2Y12 inhibitors, are needed to fully understand their combined interaction on platelet function. Furthermore, evaluation of the effects of BTKi on measurable platelet tests could provide additional clinical information on the antiplatelet effect of BTKi and ultimately serve as a decision guide for patients receiving concomitant antiplatelet therapy. Finally, prospective studies assessing BTKi bleeding risk in patients on antiplatelet therapy are needed to help elucidate their safety in this population.

Noncovalent BTKi undergoing studies in patients with hematologic malignancies demonstrated reduced risk of bleeding and, when approved, may represent a preferred alternative.^31^ In the meantime, interventional cardiologists should be able to identify patients treated with BTKi, recognize the risks of these agents, and engage in collaborative discussions with the treating hematologist to individualize care and optimize safety and outcomes.
