# Supplementary material for: Bleeding Risk With Antiplatelets and Bruton's Tyrosine Kinase Inhibitors in Patients With Percutaneous Coronary Intervention
Source: J Soc Cardiovasc Angiogr Interv. 2023 Mar 6;2(3):100608. doi: 10.1016/j.jscai.2023.100608 (PMC11308864; doi:10.1016/j.jscai.2023.100608)
Supplement: Supplemental Table S1 [file mmc1.pdf]

Supplementary Table S1. Covalent BTKi Bleeding Risk

Agent	Agency Approval	Indication	Phase 3 trial	Author et al. (year)	Study drug groups	Population	Median treatment exposure (months)	Bleeding of any grade	Major bleeding (grade≥3)	Median follow up (months)	Median PFS in months	PFS rate at specific time
Ibrutinib	FDA 2013<br>EMA 2014	Treated MCL<br>CLL/SLL<br>WM<br>MZL<br>GVHD	RESONATE	Byrd (2014)[17]	Ibrutinib	Adults with pretreated CLL/SLL	8.6	44%	1%	9.4	NR	6-mo: 88%
					Ofatumumab		5.3	12%	2%		8.1	6-mo: 65%
			RESONATE 2	Burger (2015)[18]	Ibrutinib	Adults with untreated CLL/SLL	17.4	NA	4%	18.4	NR	18-mo: 90%
					Chlorambucil		7.1	NA	2%		18.9	18-mo: 52%
Acalabrutinib	FDA 2017<br>EMA 2020	Treated MCL<br>CLL/SLL	ELEVATE-TN	Sharman (2020)[20]	Acalabrutinib plus obinutuzumab	Adults with untreated CLL	27.7	43%	2%	28.3	NR	24-mo: 93%
					Acalabrutinib		27.7	39%	2%		NR	24-mo: 87%
					Obinutuzumab plus chlorambucil		5.6	12%	0		22.6	24-mo: 47%
			ASCEND	Ghia (2020)[19]	Acalabrutinib	Adults with pretreated CLL	15.7	26%	2%	16.1	NR	12-mo: 88%
					Idelalisib Plus Rituximab		11.5 for idealisib	8%	3%		15.8	12-mo: 68%
					Bendamustine Plus Rituximab		5.6 for bendamustine	6%	3%		16.9	12-mo: 69%

BTKi, Bruton's tyrosine kinase inhibitor; CLL, chronic lymphocytic leukemia; EMA, European Medicines Agency; FDA, Food and Drug Administration; GVHD, graft versus host disease; MCL, mantle cell lymphoma; MZL, marginal zone lymphoma; NR, not reached; PFS; progression-free survival; SLL, small lymphocytic lymphoma; WM, Waldenstrom macroglobulinemia

			Acalabrutinib vs ibrutinib	Byrd (2021)[21]	Acalabrutinib	Adults with pretreated CLL	38.3	38%	5%	40.9	38.4	-
					Ibrutinib		35.5	51%	5%		38.4	-
Zanubrutinib	FDA 2019<br>EMA 2021	Treated MCL<br>Treated MZL<br>WM	SEQUOIA	Tam (2022)[22]	Zanubrutinib	Adults with untreated CLL/SLL without del(17)(p13·1)	26.07	45%	4%	26.2	NR	24 mo: 85.5%
					Bendamustine–rituximab		5.52	11%	2%		28.1	24 mo: 69.5%
					Zanubrutinib	With del(17)(p13·1)	30.0	51.4%	5%	30.5	NR	24 mo: 88.9%
			ALPINE	Brown (2022)[23]	Zanubrutinib	Adults with pretreated CLL/SLL	15	NA	3%	-	-	12 mo: 94.9%
					Ibrutinib		15	NA	4%	-	-	12 mo: 84.0%
			ASPEN	Tam (2020)[7]	Zanubrutinib	Adults with WM	18.7	4.4 per 100 person-months	6%	18.0	NR	18 mo: 85%
					Ibrutinib		18.6	7.0 per 100 person-months	9%	18.5	NR	18.5 mo: 84%

CLL, chronic lymphocytic leukemia; EMA, European Medicines Agency; FDA, Food and Drug Administration; MCL, mantle cell lymphoma; MZL, marginal zone lymphoma; NA, NR, not reached; PFS; progression-free survival; SLL, small lymphocytic lymphoma; WM, Waldenstrom macroglobulinemia
